# *Candida albicans* and candidalysin in inflammatory disorders and cancer

**DOI:** 10.1111/imm.13255

**Published:** 2020-09-13

**Authors:** Jemima Ho, Giorgio Camilli, James S. Griffiths, Jonathan P. Richardson, Nessim Kichik, Julian R. Naglik

**Affiliations:** ^1^ Centre for Host‐Microbiome Interactions Faculty of Dentistry, Oral & Craniofacial Sciences King's College London London UK

**Keywords:** *Candida albicans*, candidalysin, gut barrier, IL‐17, mucosal disease

## Abstract

As our understanding of mycology progresses, the impact of fungal microbes on human health has become increasingly evident. *Candida albicans* is a common commensal fungus that gives rise to local and systemic infections, particularly in immunocompromised patients where it can result in mortality. However, *C. albicans* has also been quietly linked with a variety of inflammatory disorders, to which it has traditionally been considered incidental; recent studies may now provide new aspects of these relationships for further consideration. This review provides a novel perspective on the impact of *C. albicans* and its peptide toxin, candidalysin, on human health, exploring their contributions to pathology within a variety of diseases.

## Graphical abstract

*Candida albicans* infections have been linked with a variety of inflammatory diseases. The ability for *C. albicans* to potentiate pathology in these instances is explored in this review.

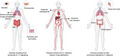

**Abbreviations**
AD: atopic dermatitis
AMPs: antimicrobial peptides
CNS: central nervous system
ECM: extracellular matrix
EGFR: epidermal growth factor receptor
EMT: epithelial–mesenchymal transition
IBD: inflammatory bowel disease
IL‐17: Interleukin
MMPs: matrix metalloproteinases
MS: multiple sclerosis
SAFS: severe asthma with fungal sensitization
VVC: vulvovaginal candidiasis

## Introduction

*Candida albicans* is a prevalent fungus that comprises part of the healthy human microbiota. Within such microbial communities, *C. albicans* often exists as a harmless commensal yeast in low‐to‐moderate numbers, likely kept in check by competing microbes and host immunity. Its ability to shift from commensal to infectious pathogen is of particular interest to the clinical understanding of *Candida* infections and remains incompletely understood. Current evidence suggests that pathogenic switching is primarily a consequence of immune compromise brought about by a variety of factors including microbial environment,^1^ immune‐suppressive drug treatment and pre‐existing infection or disease.^2^, ^3^, ^4^ Indeed, immunocompromised patients are particularly susceptible and exhibit mucosal candidiasis of enhanced severity and frequency, with potential to progress to systemic candidaemia. This represents a significant clinical burden, with ~ 2 000 000 infections in HIV + patients and 700 000 total systemic infections recorded in 2017.^5^

In addition to conditions that occur as a result of persistent or severe *Candida* infection, such as oral and vulvovaginal candidiasis (VVC) or systemic candidaemia, an increasing number of seemingly unrelated diseases have also been reported to show association with *Candida* infection. Elevated incidence of candidiasis has been linked with periodontitis,^6^, ^7^, ^8^, ^9^ inflammatory bowel disease (IBD),^10^, ^11^ and skin^12^, ^13^, ^14^ and respiratory disorders,^15^, ^16^, ^17^, ^18^ among others; however, the causal relationship in such circumstances remains unclear. Whilst weakened immunity occurring as a result of disease may certainly favour growth of opportunistic fungi, a role for *C. albicans* in perpetuating ongoing disease and promoting acute or chronic pathology may also warrant consideration, particularly in the context of its secreted toxin, candidalysin.

Candidalysin is a recently described cytolytic peptide exclusively secreted by pathogenic hyphal forms of *C. albicans*.^19^ Interestingly, candidalysin plays an important role in triggering innate antifungal immunity during infection,^20^, ^21^ which is largely governed by neutrophil and interleukin (IL)‐17 responses.^22^, ^23^, ^24^, ^25^ This review will examine the variety of diseases associated with *C. albicans* infections and assess the role of this fungus and its toxin, candidalysin, in disease development and associated pathology.

## IL‐17‐mediated disorders

The IL‐17 signalling pathway possesses critical roles in immune regulation, and aberrant function results in a range of diseases that share a common feature of chronic inflammatory‐induced pathology. This is best defined by IL‐17A, the most studied IL‐17 family member, which has been shown to contribute to dermatitis,^26^, ^27^ psoriasis,^28^ IBD,^29^, ^30^ arthritis,^31^ multiple sclerosis (MS),^32^ periodontal disease^6^, ^7^ and systemic lupus erythematosus,^33^ among other inflammatory diseases. Though much is still unknown about the mechanisms of IL‐17‐mediated pathology, its potent induction of antimicrobial peptides (AMPs), proinflammatory cytokines and downstream neutrophil recruitment^34^, ^35^, ^36^, ^37^ is thought to contribute. Of particular interest is the central role of IL‐17A in orchestration of antifungal defences. IL‐17A and associated effector molecules and cells are potently induced in response to *C. albicans* infection,^38^, ^39^, ^40^, ^41^ with candidalysin accounting for robust induction of early and innate ‘natural’ Th17 cell‐derived IL‐17A.^20^

Notably, elevated incidence or sensitization to *C. albicans* is often observed in a specific group of IL‐17‐mediated pathologies localized at mucosal and epithelial surfaces. These include periodontitis,^6^, ^7^, ^8^, ^9^ atopic dermatitis (AD),^14^, ^42^ psoriasis,^43^, ^44^ IBD,^10^, ^11^ mycotic keratitis^45^ and severe asthma with fungal sensitization (SAFS).^17^, ^18^ Moreover, evidence of improved disease outcomes following antifungal measures has been observed and suggests a role for *C. albicans* in contributing to disease pathology. Examples include tamoxifen‐induced *C. albicans* inhibition to reduce severity of periodontitis in women,^46^ as well as fluconazole treatment^47^ or faecal microbiota transplantation (inhibiting *C. albicans* burdens)^48^ to rescue ulcerative colitis symptoms in mice. There are also limited reports of improved *C. albicans*‐related respiratory diseases following fluconazole administration^15^, ^49^ (though *Aspergillus* species have been better studied in airways disease^50^, ^51^). It is likely that other examples could be found by investing more research into *C. albicans* in this context.

Furthermore, candidalysin can directly induce IL‐1β,^21^, ^52^ IL‐36^53^, ^54^ and the NLRP3 inflammasome,^55^, ^56^ central proinflammatory components known to significantly contribute to IL‐17‐mediated diseases.^57^, ^58^, ^59^, ^60^, ^61^ Together, these studies suggest a role and potential mechanisms for *C. albicans* in contributing to the elevated immune response and pathology observed in IL‐17‐mediated inflammatory diseases. However, the relationships are complex. Therapeutic blockade of IL‐17, whilst beneficial in MS,^62^ arthritis^63^ and psoriasis,^64^ does not improve AD^68^ and was shown to result in exacerbation of existing^65^, ^66^ and even de novo^67^ IBD pathology, as well as increased incidence of *Candida* infections.^29^ Greater understanding is thus required to fully delineate the complex mechanisms underlying IL‐17‐mediated diseases and how *Candida* or indeed other fungal species may impact pathology.

A significant component of successful IL‐17‐mediated antifungal response is potent neutrophil recruitment and activation,^39^, ^69^ which, at an early stage in infection, can be triggered by the presence of candidalysin.^20^ Interestingly, whilst a robust neutrophil response functions to resolve oral^21^, ^22^ and central nervous system (CNS)^25^ *C. albicans* infections, neutrophils are found to drive pathology of VVC,^52^, ^70^ *Candida* keratitis (CaK),^71^ systemic candidaemia^72^ and *C. albicans*‐associated cystic fibrosis, with the latter arising from neutrophil‐induced degradation of chitinase, suppressing host ability to protect against chitin containing *C. albicans*.^73^ These studies suggest a delicate balance and complexity of antifungal neutrophil responses, which are likely dependent on multiple components. Continued research may determine the underlying factors leading to pathology in this context.

## Barrier integrity and disease

Another component often compromised in disease and particularly in infection is the epithelium. Its integrity and function as a selectively permeable barrier are dependent on cohesive contacts between neighbouring epithelial cells. This is largely provided by E‐cadherin, a transmembrane glycoprotein that forms binding pairs with that of neighbouring cells, which, clustering together at adherens junctions, are supported by the cytoskeleton to form a tight belt across the epithelium that fastens cells together.^74^ In addition to structural defences, a healthy epithelium provides front‐line induction of innate immune responses through release of alarmins, AMPs and immune cell chemokines, all of which are additionally compromised upon loss of barrier integrity. Notably, periodontal,^75^, ^76^, ^77^ gut^78^, ^79^, ^80^ and skin^81^, ^82^, ^83^ disorders are commonly associated with the loss of E‐cadherin, resulting in enhanced barrier permeability and inflammatory pathology, with restoration of E‐cadherin showing improvement in disease outcomes.^84^

*Candida albicans* infection has also been shown to diminish E‐cadherin expression in both *in vivo* and *in vitro* infection models,^85^, ^86^, ^87^ with candidalysin highlighted as a direct contributor to epithelial damage, loss of barrier integrity and subsequent translocation of *C. albicans* across the intestinal epithelia.^88^ As the gut is considered the main site of *C. albicans* entry into the bloodstream,^89^ where it may lead to systemic candidaemia and mortality, the pathological impact of *Candida* and candidalysin at this organ appears highly significant. The ability of *C. albicans* to diminish E‐cadherin expression and other cell adhesion proteins such as occludin and desmoglein‐2,^90^ and diminish barrier integrity, may be of particular interest in the context of IBD, oral and skin disorders.

Recently, alcoholic hepatitis was found to be another potential example of the pathological impact of *C. albicans* in breaching gut barriers.^91^, ^92^ The gut–liver axis describes the relationship and role of the gut microbiome in shaping healthy liver metabolism. This connection is supported by the close anatomical proximity between the gut and liver, as well as a specialized portal circulation, which permits enhanced permeability and interaction between gut‐derived substances and liver‐resident cells. Gut‐derived bacterial components and endotoxins in particular have long been implicated in driving alcoholic liver disease.^93^, ^94^ Recent studies now show a similarly significant role for *C. albicans*. Upon infection, ligation of *C. albicans* β‐glucans to host dectin‐1 receptors on liver‐resident macrophages (kupffer cells), results in inflammatory IL‐1β release and enhanced ethanol‐induced liver disease in mice.^91^ A second, non‐dectin‐1‐mediated but candidalysin‐induced mechanism also drives elevated hepatic damage, steatosis and mortality in ethanol‐fed *C. albicans*‐infected mice.^92^ The authors additionally observe that alcoholic hepatitis patients carry elevated levels of the candidalysin‐encoding gene, *ECE1,* when compared to healthy controls. These data identify two distinct *C. albicans* mechanisms, each independently promoting alcoholic hepatitis and highlights this pathogen as a new and considerable factor for the development of alcoholic liver disease.

## Dysregulated growth signalling

The ability of *C. albicans* to activate the epidermal growth factor receptor (EGFR) may also contribute to *C. albicans*‐associated comorbidities. The EGFR is a transmembrane protein with a broad range of functions controlling various cell proliferative and maintenance roles, in addition to immune induction. It is often found highly dysregulated, via overexpression or constitutive activation, in a select group of cancers including head and neck, breast, lung, colon and vulvovaginal cancers.^95^ Interestingly, the majority of these EGFR‐associated cancers are located at sites where *C. albicans* commonly infects, with reports providing contrasting evidence both for and against elevated incidences of candidiasis in these patients. Whilst immune suppression resulting from anticancer therapy may indeed play a role, a long‐standing debate on the ability of *C. albicans* to potentiate oncogenic disease exists, primarily in oral cancer. Recent studies may now provide additional aspects for consideration.^96^

*C. albicans* infection potently activates the EGFR. Upon cell adhesion, EGFR is bound and activated by the fungal cell wall protein Als3p, which initiates endocytosis of the fungus, providing an entry mechanism into host cells.^97^ Additionally, candidalysin can indirectly activate EGFR through a complex mechanism involving matrix metalloproteinases (MMPs) and EGFR ligands, resulting in downstream immune activation.^21^ Notably, MMPs^98^ and EGFR ligands^95^ are each independently implicated in a number of cancers. Other observations suggesting contribution to cancer development include the ability for *C. albicans* to activate epithelial MAPK^99^ and ERK signalling pathways, which are associated with growth and proliferation; loss of E‐cadherin and occludin,^90^ observed in epithelial–mesenchymal transition (EMT); activation of angiogenesis^100^ and pro‐angiogenic factors;^101^, ^102^ and the ability of *Candida* to enhance production of known carcinogenic molecules such as nitrosamines^103^, ^104^ and acetaldehyde.^105^, ^106^ However, clinical and *in vivo* evidence substantiating a direct causal or potentiating role for *C. albicans* in cancer is particularly limited. As such, the association here remains ambiguous.

Activation of MMPs is also observed in oral disease,^107^ resulting in breakdown of gingival and periodontal ligament collagens, tissue remodelling, inflammation and uncontrolled extracellular matrix (ECM) turnover, also associated with cancer.^108^, ^109^ Investigation into potential links with *C. albicans* would be of great interest given the known associations of this fungus with oral disease, its ability to signal through^21^ and induce MMPs,^110^ and MMP activation being a demonstrated mechanism for disease utilized by other oral pathogens.^111^, ^112^

## Conclusions

**Figure 1:**
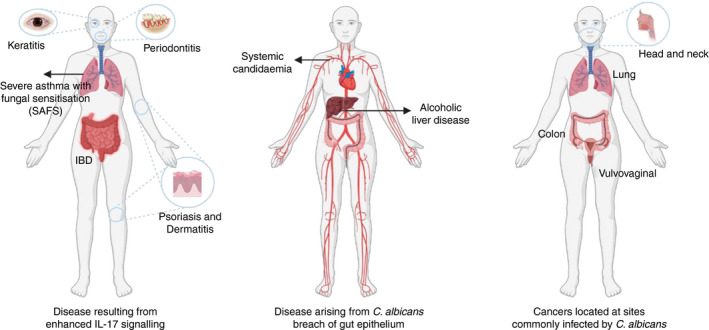
*Candida albicans* potential contribution to disease. Potential *C. albicans* mechanisms of contributing to disease include potent induction of IL‐17 signalling, breach of gut epithelial barriers and activation of multiple cancer‐associated factors.

As we increase our understanding of *C. albicans* induced pathophysiology, the potential for infection to contribute to several comorbidities becomes increasingly apparent. Its natural distribution throughout the body and ability to activate events highly linked with disease may be of significant consequence. Induction of IL‐17‐mediated signalling, breach of epithelial barriers and activation of cancer‐associated factors provide the most convincing examples of its ability to contribute to disease (summarized in Fig. 1), though greater understanding is required to fully delineate its role in these instances, as well as others, yet unknown. Further research into the association of *C. albicans* with these diseases shall undoubtedly shed light on new mechanisms of disease development, which may shift perceptions of this under‐investigated microbe and its influence on human health.

## Disclosures

The authors declare no conflict of interest.

## Data Availability

Not applicable. No data were generated in the making of this article.
